# Research Progress on the Chemical Composition, Pharmacological Effects and Veterinary Clinical Application of *Purslane* (*Portulaca oleracea* L.)

**DOI:** 10.3390/vetsci13101017

**Published:** 2026-09-26

**Authors:** Jianming Ren, Peijin Song, Lan Zhang, Pengfei Zhao, Shiyu Tao, Jiancheng Jiang, Fanlin Wu, Xinsheng Lu, Peng Ji

**Affiliations:** 1 College of Chemistry and Life Sciences, Gansu Normal University for Nationalities, Hezuo 747000, China; 2 College of Veterinary Medicine, Gansu Agricultural University, Lanzhou 730070, China; 15095459318@163.com; 3 Lanzhou Institute of Husbandry and Pharmaceutical Sciences, Chinese Academy of Agricultural Sciences, Lanzhou 730050, China

**Keywords:** *Portulaca oleracea*, chemical composition, pharmacological action, veterinary clinical application

## Abstract

Veterinary practice faces rising antimicrobial resistance, driving interest in plant-derived alternatives. *Purslane* (*Portulaca oleracea* L.), a medicinal edible herb with antimicrobial, anti-inflammatory, and immunomodulatory properties, is a promising candidate. This narrative review synthesizes 160 studies into a unified framework linking chemical composition (flavonoids, alkaloids, polysaccharides, organic acids), pharmacological activities (12 major effects), and veterinary applications. Mechanistically, polysaccharides act as immunomodulators via the TLR4/MyD88 pathway; flavonoids (quercetin, kaempferol) exert antioxidant/anti-inflammatory effects through NF-κB/MAPK inhibition and Nrf2 activation; organic acid extracts show potent anti-MRSA activity (MIC 0.5–1.0 mg/mL); and alkaloids enhance insulin secretion. In vivo, dietary purslane at 2–3% improves broiler growth performance and feed conversion, while supplementation enriches omega-3 fatty acids in eggs and meat. However, translational barriers persist, including limited pharmacokinetic data, lack of large-scale field trials, and insufficient safety and residue-depletion information for food-producing animals. We outline future priorities, including species-specific pharmacokinetic studies, field efficacy trials, and quality-control standards, positioning purslane as a mechanism-rich, antibiotic-sparing candidate for further investigation in veterinary therapeutics and functional feed additives.

## Simple Summary

Purslane is a medicinal plant with antibacterial, anti-inflammatory, and immune-boosting properties. This review examines its potential as a natural alternative to antibiotics in livestock and companion animal care. Dietary purslane has been reported to improve growth in broilers, enrich eggs and meat with omega-3 fatty acids, and help reduce diarrhea in piglets. Despite promising results, more research is needed on proper dosing, field effectiveness, and safety in food-producing animals. This work highlights purslane as a promising candidate for reducing antibiotic dependence in veterinary practice.

## 1. Introduction

*Purslane* (*Portulaca oleracea* L.) is an annual succulent herb of the Portulacaceae family, widely distributed in temperate and tropical regions worldwide. Its dried aerial parts are known in traditional Chinese medicine as *Portulacae Herba* and have been used for centuries in human medicine for various indications, including dysentery, eczema, and hemorrhoidal bleeding [1]. In traditional Chinese veterinary medicine, purslane has also been employed for gastrointestinal disorders and inflammatory conditions in livestock [2]. In recent decades, growing concerns over antimicrobial resistance and the demand for alternatives to in-feed antibiotics have driven renewed interest in phytogenic feed additives, positioning purslane as a promising candidate for veterinary applications [3,4]. Modern phytochemical research has identified a diverse array of bioactive compounds in purslane, including flavonoids, alkaloids, polysaccharides, organic acids, and omega-3 fatty acids [4]. These constituents exhibit a broad spectrum of pharmacological activities, such as anti-inflammatory, antibacterial, antioxidant, immunomodulatory, and metabolism-regulating effects. However, the available evidence spans a wide range of experimental models—from in vitro assays and rodent studies to human clinical trials and a limited number of trials in target veterinary species—and these different levels of evidence are often presented interchangeably in the existing literature. This conflation creates a misleading impression that effects demonstrated in non-target models have been validated in livestock or companion animals.

To date, a comprehensive and critical synthesis that explicitly distinguishes between (i) in vitro bioactivity, (ii) rodent pharmacological effects, (iii) human clinical data, and (iv) evidence from target veterinary species is lacking for purslane. The present narrative review addresses this gap by systematically collating and critically appraising the available evidence on purslane’s chemical composition, pharmacological mechanisms, and veterinary applications. We adopt a hierarchical evidence framework to clearly differentiate the strength and translational relevance of findings derived from different experimental systems. Particular attention is paid to safety considerations, including oxalate and nitrate content, which have been linked to poisoning incidents in ruminants. Our goal is to provide veterinarians and animal scientists with a balanced, evidence-based resource that highlights both the potential and the limitations of purslane in animal health and production, and to identify priority areas for future research.

## 2. Literature Search Strategy

Searches were performed in PubMed, Web of Science, CNKI, and Wanfang from inception to May 2026, using database-specific free-text terms and, where applicable, publication-type filters. The complete database-specific search strategies are provided in Supplementary Table S1. Two reviewers independently screened titles/abstracts and full texts. After removing duplicates, refining by subject category (e.g., pharmaceutical/analytical chemistry for compositional studies; pharmacology/molecular biology for mechanistic studies; agronomy/animal science for applied studies), and excluding reviews without primary data, conference abstracts, and non-English/non-Chinese papers, we retained 160 eligible studies (129 English, 31 Chinese). The category numbers were: overview/ethnopharmacology (24), chemical composition/analysis (44), pharmacological mechanisms (63), and agricultural/feed/toxicology (29). No quantitative synthesis or formal risk-of-bias assessment was performed due to study heterogeneity and the narrative scope of the review.

## 3. Results

### 3.1. Chemical Composition

**Figure 1:**
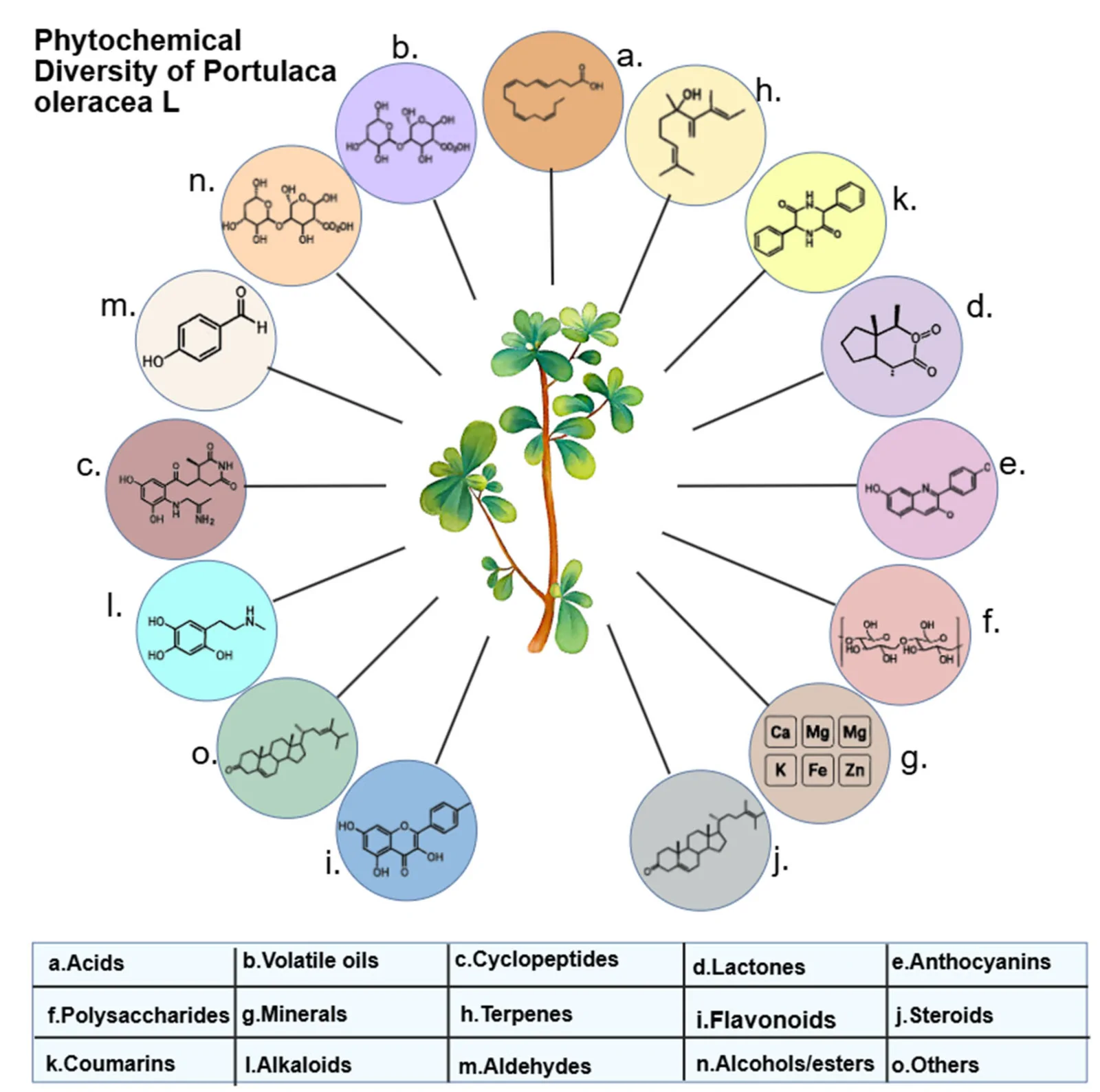
The main chemical constituents of *Portulacae Herba.* The diagram summarizes the main categories of bioactive phytochemicals isolated from *Portulacae Herba*. Representative chemical structures are presented for each group: (**a**) acids, (**b**) volatile oils, (**c**) cyclopeptides, (**d**) lactones, (**e**) anthocyanins, (**f**) polysaccharides, (**g**) minerals, (**h**) terpenes, (**i**) flavonoids, (**j**) steroids, (**k**) coumarins, (**l**) alkaloids, (**m**) aldehydes, (**n**) alcohols/esters, (**o**) others.

Purslane harbors an extensive array of chemical constituents, mainly comprising flavonoids, alkaloids, polysaccharides, organic acids, essential oils, cyclic dipeptides, minerals, and vitamins, as illustrated in Figure 1. Given that this review is oriented toward veterinary applications, the following subsections emphasize those compound classes with established or promising bioactivities relevant to animal health and production.

#### 3.1.1. Flavonoids

Flavonoids represent one of the predominant active components contributing to the pharmacological properties of purslane [5]. Accumulating evidence has further confirmed that flavonoids are the most abundant and biologically active secondary metabolites in purslane [6,7], with quercetin, kaempferol, and other flavonoid derivatives as the primary constituents [7]. These compounds exert prominent antioxidant, anti-inflammatory, hypoglycemic, and lipid-regulating bioactivities [8]. The specific content of flavonoids in purslane is influenced by the origin [9], harvest season [10], and extraction method [11]. From a veterinary perspective, flavonoid-rich extracts have demonstrated particular promise in mitigating inflammatory conditions in poultry and modulating lipid metabolism in livestock. The major flavonoids in purslane are shown in Table 1.

#### 3.1.2. Alkaloids

Over 20 types of alkaloids have been isolated and identified from purslane, mainly including betaine, betacyanin, and betaflavin, as well as novel amide-type and indole-type alkaloids [17]. These alkaloids possess anti-inflammatory, antioxidant, anticholinesterase, and neuroprotective properties [18]. Of particular veterinary interest, certain alkaloids have demonstrated insulin-secretion-enhancing effects, suggesting potential applications in managing metabolic disorders in companion animals. The major alkaloid constituents of purslane are listed in Table 2.

#### 3.1.3. Polysaccharides

Polysaccharides are an important class of bioactive macromolecules in purslane; however, the polysaccharide content obtained by researchers from purslane extracts varies significantly [25,26]. Purslane polysaccharides are mainly composed of neutral and acidic sugars, with the primary monosaccharide units including galacturonic acid (GalA), arabinose (Ara), galactose (Gal), rhamnose (Rha), glucose (Glc), and xylose (Xyl) [26]. Notably, polysaccharides are the principal immunomodulatory components of purslane, exerting their effects through activation of the TLR4/MyD88 pathway and promotion of macrophage polarization. This property is particularly relevant to veterinary applications, including enhancement of vaccine efficacy and mitigation of weaning stress in piglets. Furthermore, purslane polysaccharides exhibit multiple bioactivities, including immune enhancement, and antioxidant, hypoglycemic, anti-inflammatory, and gut microbiota-modulating effects [27].

#### 3.1.4. Organic Acid Compounds

A wide variety of organic acids have been identified in purslane, primarily falling into three categories: aliphatic dicarboxylic and tricarboxylic acids, aromatic organic acids and their derivatives, and novel organic acids and derivatives with unique structures [28]. Among these, oxalic acid, malic acid, and citric acid are the most abundant organic acids [29]. The organic acid compounds in purslane exert anti-inflammatory, antioxidant, antibacterial, antiviral, metabolism-regulating, and cardiovascular-protective effects through multi-target, multi-pathway mechanisms [30]. Critically, organic acid extracts have demonstrated potent antibacterial activity against methicillin-resistant *Staphylococcus aureus* (MRSA) with minimum inhibitory concentrations (MICs) of 0.5–1.0 mg/mL, highlighting their potential as natural antimicrobial alternatives in livestock production. Table 3 lists the major organic acid compounds found in purslane.

Purslane is rich in polyunsaturated fatty acids (PUFAs), particularly those in the omega-3 and omega-6 series [37]. The fatty acid compositions and proportions vary by plant part; when considered as a whole, the entire purslane plant consists primarily of linolenic acid (47.16%), linoleic acid (22.00%), and palmitic acid (17.4%) [38], while the seeds are dominated by linoleic acid (45.86%) and alpha-linolenic acid (30.16%) [37]. Spyridon A. Petropoulos and colleagues found that the edible parts of purslane generally contain omega-6 and omega-3 fatty acids; the leaves have a higher alpha-linolenic acid content than the stems, while the stems primarily contain palmitic acid and linoleic acid [39]. These are key substances responsible for purslane‘s anti-inflammatory, antioxidant, immune-modulating, cardiovascular-protective, and neuroprotective effects. From a veterinary nutrition standpoint, the omega-3 enrichment in eggs and meat from poultry fed purslane-supplemented diets represents a valuable functional food application.

#### 3.1.5. Minerals and Vitamins

Minerals are an essential and important class of nutrients for animals and play a crucial role in their health. Minerals found in purslane include copper, iron, manganese, magnesium, potassium, calcium, and phosphorus [40], with potassium (494 mg/100 g), magnesium (68 mg/100 g), and calcium (65 mg/100 g) being particularly abundant [41]. Purslane is rich in various essential vitamins, including vitamin C, vitamin A, vitamin E, and B vitamins [9,20]. K. Makangali et al. [42] found that purslane contains high levels of B vitamins, such as thiamine (B_1_), riboflavin (B_2_), niacin (B_3_), pantothenic acid (B_5_), and pyridoxine (B_6_) [10]. These micronutrients contribute to the nutritional value of purslane as a feed supplement, particularly for improving the mineral status of livestock and poultry.

#### 3.1.6. Essential Oils and Other Bioactive Components

Plant essential oils are a class of volatile, water-insoluble oily liquids found in plants that exhibit strong antimicrobial activity [43]. Liu Pengyan [44] and colleagues found that the essential oil components in purslane include linalool, norepinephrine, linolenic acid esters, 3, 7, 11, 15-tetramethyl-2-hexadecenol, yak-ol, and heptadecane. Qiao Zhiwen [45] and colleagues used silica gel column chromatography and HPLC to isolate compounds such as rye grassin, linoleic acid triglycerides, and α-palmitic acid monoglycerides from purslane; among these, rye grassin was isolated from purslane for the first time.

Additional trace bioactive components identified in purslane primarily include cyclo(phenylalanine-tyrosine), cyclo(alanine-leucine), cyclo(alanine-isoleucine), cyclo(tyrosine-leucine) [20], cyclo(phenylalanine-isoleucine), and cyclo(tyrosine-alanine) [46]. Lactone compounds (coumarins, sesquiterpene lactones, hydroxydihydrobofuvonlactone, rye grass lactone, isorye grass lactone, and dehydrodipropyl lactone) [47] have demonstrated promising effects in anti-inflammatory, antitumor, antibacterial, antifungal, anticholinesterase, and neuroprotective activities [48], as well as anthocyanins (purslanein, acetylbetanin, betanin 5-O-β-fibrodisaccharide, and isobetanin 5-O-β-fibrodisaccharide) [49].

The terpenoids and sterols found in purslane primarily include lupeol, betulinic acid, ursolic acid, luteolin, parketol, cycloartanol, glycyrrhetinic acid, oleanolic acid, and carotene, among others. Youngwan Seo [50] et al. isolated purslane monoterpene B, (3S)-3-O-(α-D-glucopyranosyl)-3, 7-dimethyl-oct-1, 5-diene-3, 7-diol, and (3S)-3-O-(α-D-glucopyranosyl)-3, 7-dimethyl-oct-1, 6-diene-3-ol. Li Bo [51] detected 4α-methyl-3β-hydroxy-suberan, sitosterol glucoside, and β-sitosterol in purslane. Yang Qi [52] detected two compounds in purslane: 3-acetyl-tungic acid and suberone.

Coumarin compounds are a class of natural secondary metabolites widely found in the plant kingdom [53]. Multiple coumarin compounds have been isolated and characterized from purslane in existing studies [54], with representative constituents including bergapten [53,55], umbelliferone, scopolin, isoanise, eucalyptol, lonchocarpic acid, lonchocarpenin, 6, 7-dihydroxycoumarin, and trans-p-coumaric acid [31].

#### 3.1.7. Aldehydes, Alcohols, and Esters

Purslane contains abundant aldehydes, alcohols, and esters, with considerable differences in their individual contents [56]. Volatile aldehydes, alcohols, and esters occupy a very low proportion of the whole-plant dry weight of purslane, whereas non-volatile structural esters (e.g., phenolic acid esters) are present at relatively higher levels [21]. These aldehydes, alcohols, and esters exert antibacterial, anti-inflammatory, antioxidant, and neuromodulatory effects via multi-target and multi-pathway synergistic mechanisms [57]. The major alcohols identified in purslane include inositol, n-hexacosanol, β-amyrin, and lupeol [58]; while the predominant esters consist of methyl 4-hydroxyphenylacetate, dimethyl L-malate, and 1-methyl L-citrate. Additionally, distinctive bioactive compounds including portulacaside A and portulacaside B, which belong to the amide and glycoside categories, have been isolated and identified from purslane [59]. Multiple trace active components, such as eugenin and β-sitosterol, have also been detected, further diversifying the chemical profile of purslane. The alcohol and ester constituents of purslane are summarized in Table 4. While these compounds contribute to the overall pharmacological profile of purslane, their concentrations are generally low and their specific contributions to veterinary therapeutic effects require further investigation.

### 3.2. Pharmacological Effects

Purslane exhibits a broad spectrum of pharmacological activities, including antitussive and anti-asthmatic, anti-inflammatory, antibacterial, antiviral, cardioprotective, lipid-lowering, hepatoprotective, neuroprotective, hypoglycemic, antitumor, antioxidant, immunomodulatory, and digestive system regulatory effects. Among these, the anti-inflammatory, immunomodulatory, antibacterial, and growth-promoting effects are most relevant to veterinary applications and are therefore emphasized in the following sections.

#### 3.2.1. Core Biological Activities

##### Antioxidant Activity

Oxidative stress and excessive accumulation of free radicals are key factors contributing to tissue damage and aging. Purslane exerts antioxidant effects by scavenging free radicals, activating endogenous antioxidant enzymes, and regulating the Nrf2/ARE pathway, with polysaccharides, flavonoids, phenolics, and alkaloids serving as its primary active components [62,63]. Dried purslane powder can reduce malondialdehyde levels in the body, enhance SOD activity, and alleviate lipid peroxidation damage [64]. The antioxidant activity of phenolics, alkaloids, and flavonoid glycosides in raw purslane is superior to that in processed purslane; among these, quercetin and kaempferol glycosides can efficiently scavenge free radicals through dual pathways [65,66]. Total flavonoids from purslane protect cell membranes from free radical damage; its extract significantly enhances the body’s antioxidant enzyme activity and reduces the levels of oxidative products, and the isoflavones it contains possess excellent free radical scavenging capacity [67]. Flavonoids, polysaccharides, and alkaloids can effectively scavenge various free radicals and inhibit lipid peroxidation [68].

##### Anti-Inflammatory and Immunomodulatory Activities

Anti-inflammatory activity represents the most thoroughly investigated and well-supported pharmacological property of purslane. This activity acts through multiple molecular targets and signaling pathways, mainly mitigating systemic inflammatory injury by suppressing the secretion of pro-inflammatory mediators and interrupting inflammatory cascades [69]. Purslane alkaloids suppress nitric oxide (NO) production and downregulate the expression of pro-inflammatory factors including TNF-α and IL-6 in a dose-dependent fashion, which attenuates inflammatory reactions [70]. Chang et al. isolated anti-inflammatory oligopeptides from purslane that markedly inhibit LPS-stimulated inflammatory responses in macrophages, and the anti-inflammatory potency of these peptides is tightly associated with their amino acid sequences [71]. He et al. reported that purslane ethanol extract alleviates zymosan-induced joint inflammation in mice in a dose-dependent manner, relieving joint swelling and inflammatory infiltration. The extract elicits anti-inflammatory and analgesic effects via modulation of the Nrf2 signaling pathway [72]. In vivo animal experiments have verified that purslane extract reduces inflammatory ear edema in mice. High doses of purslane polysaccharides effectively restrain tissue edema and excessive granulation tissue formation, exhibiting robust anti-inflammatory activities [73].

**Figure 2:**
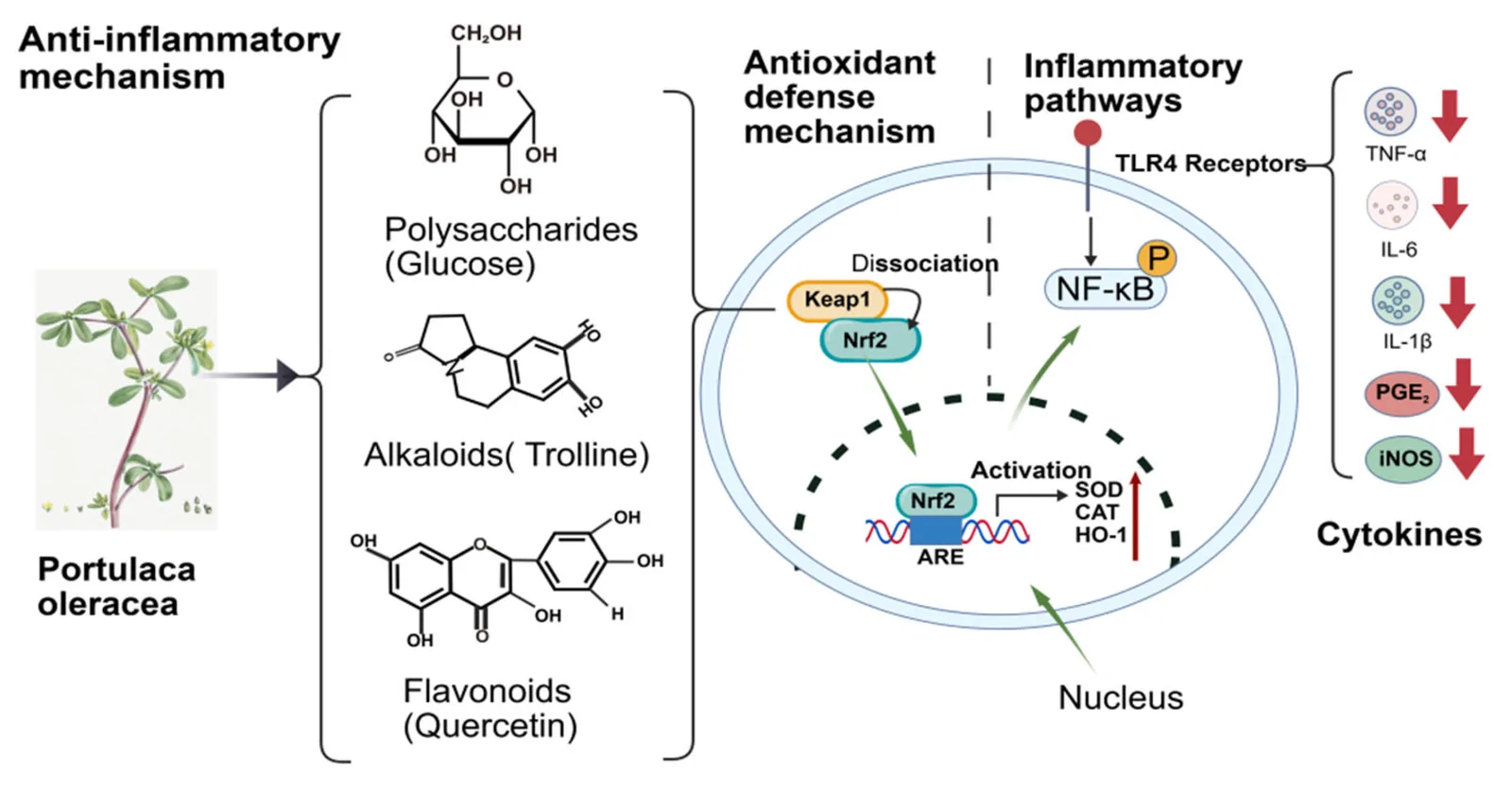
Anti-inflammatory mechanism of *Portulacae Herba.* Representative bioactive components (polysaccharides, trolline-type alkaloids, quercetin-type flavonoids) from Portulaca oleracea L. activate the Keap1-Nrf2/ARE antioxidant pathway and inhibit the TLR4/NF-κB-mediated inflammatory pathway. The green arrows indicate activation or promotion of molecular events, and the red downward arrows represent reduction in the levels of pro-inflammatory mediators (TNF-α, IL-6, IL-1β, PGE_2_, iNOS). Abbreviations: TLR4, toll-like receptor 4; NF-κB, nuclear factor kappa-light-chain-enhancer of activated B cells; Keap1, Kelch-like ECH-associated protein 1; Nrf2, nuclear factor erythroid 2-related factor 2; ARE, antioxidant response element; SOD, superoxide dismutase; CAT, catalase; HO-1, heme oxygenase-1.

The omega-3 unsaturated fatty acids in purslane promote the synthesis of protective prostaglandins, inhibit the release of pro-inflammatory mediators, and regulate the progression of local inflammation [74]. Its polysaccharides can block IL-6 secretion and receptor binding, downregulate the expression of genes and proteins associated with intestinal inflammation, and inhibit the amplification of the inflammatory cascade [75]. A decoction of purslane can also reduce serum IgE levels in patients with atopic dermatitis, alleviate inflammatory skin damage, and exhibit good anti-inflammatory effects in vivo [76]. Mechanistic studies indicate that purslane exerts anti-inflammatory effects by inhibiting the NF-κB and MAPK pathways, downregulating NLRP3 inflammasome activity, and blocking the NF-κB/STAT3 signaling axis [72]; it can prevent the transformation of chronic inflammation into cancer and also holds potential value in protecting against radiation-induced inflammation (Figure 2). Currently, most relevant studies focus on validating single pathways, lacking systematic analysis of multi-pathway interactive regulation, and in vivo experimental evidence remains to be supplemented [77].

Purslane primarily enhances systemic immune function by promoting immune organ development, activating immune cells, and modulating the immune microenvironment; polysaccharides serve as its core bioactive constituents [78,79]. It facilitates the development and maturation of the thymus and spleen and activates innate immune responses. Purslane polysaccharides promote lymphocyte proliferation, regulate T-cell subset distribution, and support immune system development and maturation [80,81]. At the same time, purslane polysaccharides can regulate macrophage polarization via the TLR4/MyD88 pathway, promoting the differentiation of anti-inflammatory macrophages and accelerating the resolution of inflammation [82,83]. Purslane polysaccharides also elevate lymphocyte counts, enhance systemic immune cytotoxicity, maintain immune homeostasis, and attenuate immune evasion [84,85]. These polysaccharides exhibit favorable adjuvant activity by promoting specific antibody production and mediating balanced immune responses, demonstrating potential for development as vaccine carriers [86,87]. Additionally, they enhance NK cell and macrophage activity, augment nonspecific immune function, and concurrently promote intestinal sIgA secretion to reinforce the mucosal immune barrier [88,89].

##### Antibacterial and Antiviral Effects

Purslane has long been utilized for the treatment of dysentery and cutaneous infections in traditional practice. Modern research has validated its broad-spectrum antibacterial activity; it suppresses the proliferation of most Gram-positive and Gram-negative bacteria through disrupting bacterial cell membranes, hindering biofilm formation, and interfering with quorum-sensing systems [90]. Liu et al. revealed that organic acid extracts from purslane exert prominent in vitro and in vivo inhibitory effects against methicillin-resistant *Staphylococcus aureus* (MRSA), with minimum inhibitory concentrations (MICs) ranging from 0.5 to 1.0 mg/mL. The extract damages bacterial structures, inhibits biofilm development, and facilitates wound repair in mice suffering from skin infections [91]. Although purslane displays moderately weaker antibacterial potency against Shigella species relative to synthetic antimicrobials, it can effectively retard the emergence of bacterial resistance, and its molecular targets differ substantially from those of conventional antibiotics [92]. Lei et al. [93,94] isolated numerous antibacterial active constituents from purslane and clarified its antimicrobial mechanisms at the molecular level. Organic acids, flavonoids, and polyphenols constitute the principal antimicrobial compounds within purslane, marking this herb as a promising natural, green antimicrobial resource [91,94]. Additional investigations have demonstrated that purslane polysaccharides and polyphenols possess inhibitory effects on diverse enveloped viruses [95].

However, the antiviral evidence remains preliminary and is predominantly derived from in vitro assays. To date, only a limited number of viral species (e.g., porcine rotavirus) have been tested, and the effective concentrations, mechanisms of action, and in vivo efficacy in target animal species have not been systematically established. Therefore, the antiviral potential of purslane should be interpreted with caution, and further studies using relevant veterinary viral pathogens and appropriate animal models are urgently needed.

#### 3.2.2. Effects on Organ Systems and Metabolism

##### Respiratory System Effects

Modern investigations have verified that purslane extract exhibits potent antitussive, bronchodilatory, and airway-protective activities, endowing it with prominent research and clinical prospects [8]. Pharmacological analyses reveal that purslane extract produces antitussive and bronchodilatory actions via activating β-adrenergic receptors within tracheal smooth muscle, which further relieves bronchial smooth muscle spasm [60,96]. Additional reports demonstrate that purslane extract effectively mitigates bronchial smooth muscle spasms triggered by external irritants, alleviates wheezing, and markedly suppresses ammonia-evoked cough reflex, confirming its favorable antitussive and bronchodilatory efficacy [96]. Existing evidence also suggests that purslane facilitates airway dilation through competitive antagonism of muscarinic receptors and modulation of calcium ion channels. Its flavonoid constituents, represented by quercetin, are capable of inhibiting mast cell degranulation and decreasing histamine secretion, consequently attenuating airway inflammation and ameliorating allergic cough and wheezing [97]. Several other studies have validated the respiratory protective potential of purslane; nevertheless, dedicated mechanistic explorations in this field remain insufficient [98]. Currently, most findings concerning the antitussive and anti-wheezing properties of purslane are derived from review summaries and indirect deductions. Moreover, purslane is mostly examined as an ingredient in compound preparations in relevant studies, rendering it challenging to attribute the observed therapeutic effects solely to purslane. Direct experimental evidence from in vitro airway smooth muscle assays and asthma animal models is still lacking. Future studies should construct standardized allergic asthma and cough-induced animal models to systematically elucidate its mechanisms targeting β_2_-adrenergic receptors and muscarinic receptors, so as to further improve the pharmacodynamic theoretical basis underlying its antitussive and bronchodilatory effects [99].

##### Cardiovascular and Lipid-Regulating Effects

**Figure 3:**
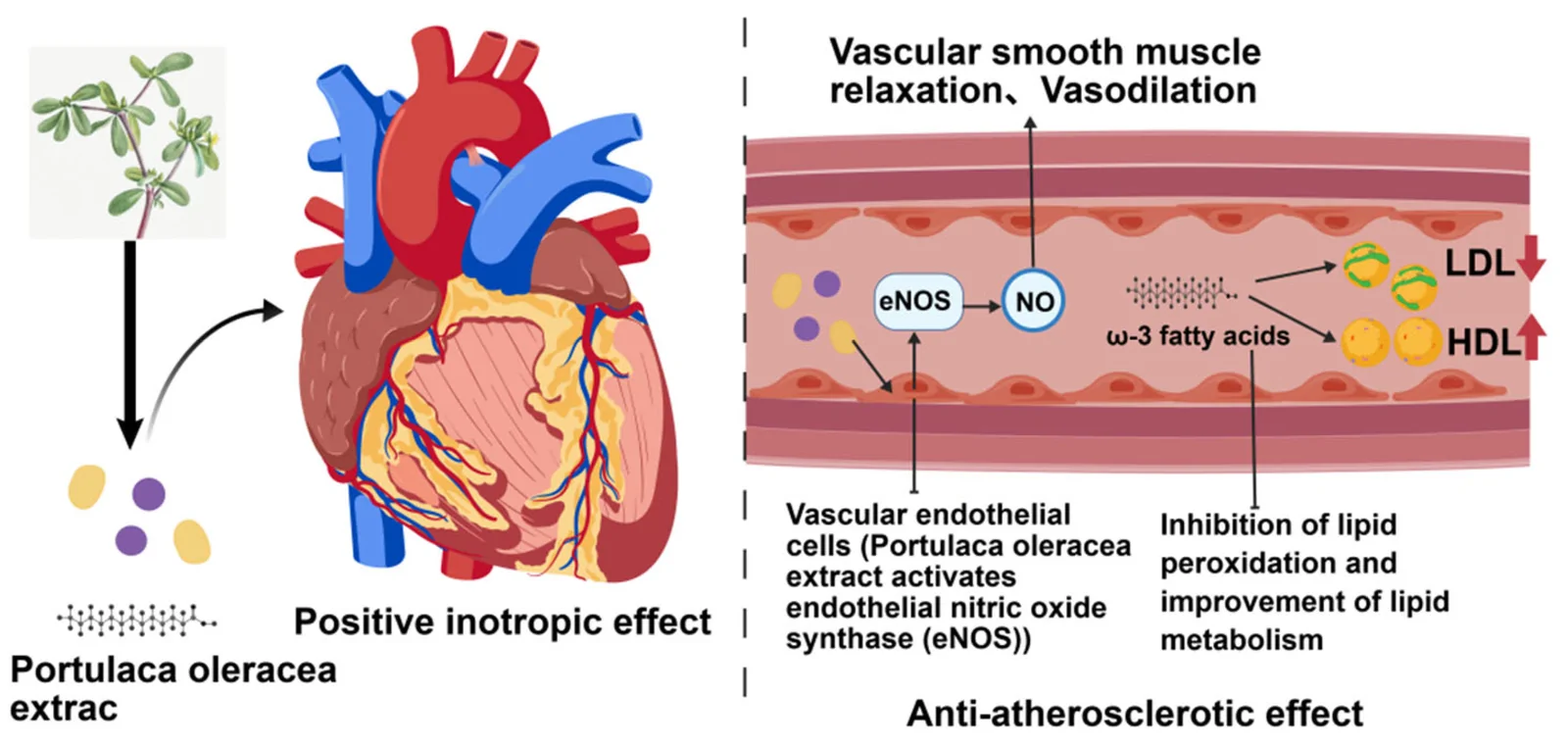
Effects of *Portulacae Herba* on the cardiovascular system and blood lipids. *Portulaca oleracea* L. extract exerts a positive inotropic action on the myocardium. It activates eNOS-mediated NO release in vascular endothelial cells, which induces vascular smooth muscle relaxation and vasodilation. The ω-3 fatty acids from Portulaca oleracea inhibit lipid peroxidation, reduce low-density lipoprotein (LDL, red downward arrow) and elevate high-density lipoprotein (HDL, red upward arrow), thereby contributing to anti-atherosclerotic effects. Abbreviations: eNOS, endothelial nitric oxide synthase; NO, nitric oxide; LDL, low-density lipoprotein; HDL, high-density lipoprotein.

Purslane is abundant in bioactive constituents including omega-3 fatty acids and flavonoids. It confers multi-targeted cardioprotective effects via antioxidant and anti-inflammatory activities, modulation of lipid metabolism, and suppression of the renin–angiotensin system, accompanied by favorable pharmacological safety and limited adverse effects [100]. Purslane decoction displays cardiotonic properties that strengthen myocardial contractility. In comparison with epinephrine, it possesses a wider therapeutic index without cardiotoxicity, and is capable of repairing injured myocardium and retarding the progression of cardiac dysfunction [101]. Existing studies have verified that purslane extract alleviates myocardial oxidative injury as well as disturbances in blood pressure and heart rate induced by hyperthyroidism and diabetes. It restores the activity of endogenous antioxidant enzymes and inhibits angiotensin-converting enzyme, thereby producing hypotensive and cardioprotective outcomes [102]. Meanwhile, purslane improves vascular endothelial function, modulates serum lipid profiles and blood pressure, and attenuates cardiovascular injuries linked to metabolic syndrome through AMPK activation and regulation of the PPAR-γ signaling pathway [103]. Furthermore, endogenous omega-3 fatty acids in purslane restrain cholesterol absorption, maintain vascular homeostasis, and lower the risk of cardiovascular disorders (Figure 3).

Hyperlipidemia frequently triggers systemic lipid metabolism disorders. Rich in omega-3 fatty acids and flavonoids, purslane effectively modulates blood lipid profiles and ameliorates aberrant lipid metabolism [104]. In vivo animal assays demonstrate that purslane homogenate regulates serum lipid concentrations in rats fed a high-fat diet, elevates hepatic antioxidant capacity, and mitigates high-fat diet-evoked hepatocyte injury [105,106]. Purslane seeds markedly decrease serum triglyceride, total cholesterol, and low-density lipoprotein cholesterol levels to alleviate dyslipidemia [100,107]. Such effects are realized via suppression of key enzymes responsible for hepatic cholesterol biosynthesis and stimulation of bile acid excretion, which collectively improve multiple serum lipid biomarkers [108]. Moreover, purslane flavonoids, predominantly quercetin and kaempferol glycosides, inhibit pancreatic lipase activity and reduce intestinal absorption of dietary lipids. Meanwhile, abundant α-linolenic acid (an omega-3 fatty acid) present in purslane seed oil contributes to the modulation of systemic lipid metabolism [109] (Figure 3).

##### Hepatoprotective Effects

Purslane exhibits significant hepatoprotective activity and can mitigate chemical and metabolic liver damage through antioxidant and anti-inflammatory effects, as well as by regulating lipid metabolism and signaling pathways [110]. Purslane water extract can reduce abnormal serum markers such as AST, ALT, and ALP in rats with liver injury models, alleviate pathological liver damage, effectively relieve hepatic steatosis, and intervene in fatty liver disease [111]. At the same time, it can activate the AMPK pathway, inhibit the activity of key enzymes involved in lipid synthesis, reduce fat accumulation in the liver, and improve hepatic steatosis [112]. Purslane can downregulate the expression of inflammatory factors such as IL-6 and TNF-α, thereby inhibiting oxidative stress and inflammatory damage in the liver [113]. Its total flavonoids can suppress the expression of TGF-β1, a key factor in liver fibrosis, improve liver fibrosis, and prevent the development of cirrhosis and liver cancer, while also alleviating radiation-induced liver damage [114].

##### Neuroprotective Effects

The flavonoids, phenolic acids, alkaloids, and unsaturated fatty acids found in purslane can protect the nervous system through antioxidant and anti-apoptotic effects, as well as by regulating neurotransmitter balance [115,116]. Its flavonoid components can inhibit acetylcholinesterase activity and optimize nerve conduction, suggesting a potential role in the treatment of Alzheimer‘s disease [56]. Purslane water extracts can upregulate central GABA levels and exert anticonvulsant effects, while ethanol extracts can alleviate brain hypoxia-induced damage and protect neural tissue [117]. Seed extracts can improve behavioral abnormalities caused by neurotoxicity, balance neurotransmitters in brain tissue, and inhibit neuronal apoptosis by regulating the Bax/Bcl-2 ratio [118,119]. Current research is largely limited to acute neurological injury models, and there remain gaps in studies on various chronic neurodegenerative diseases.

##### Hypoglycemic and Metabolic Effects

**Figure 4:**
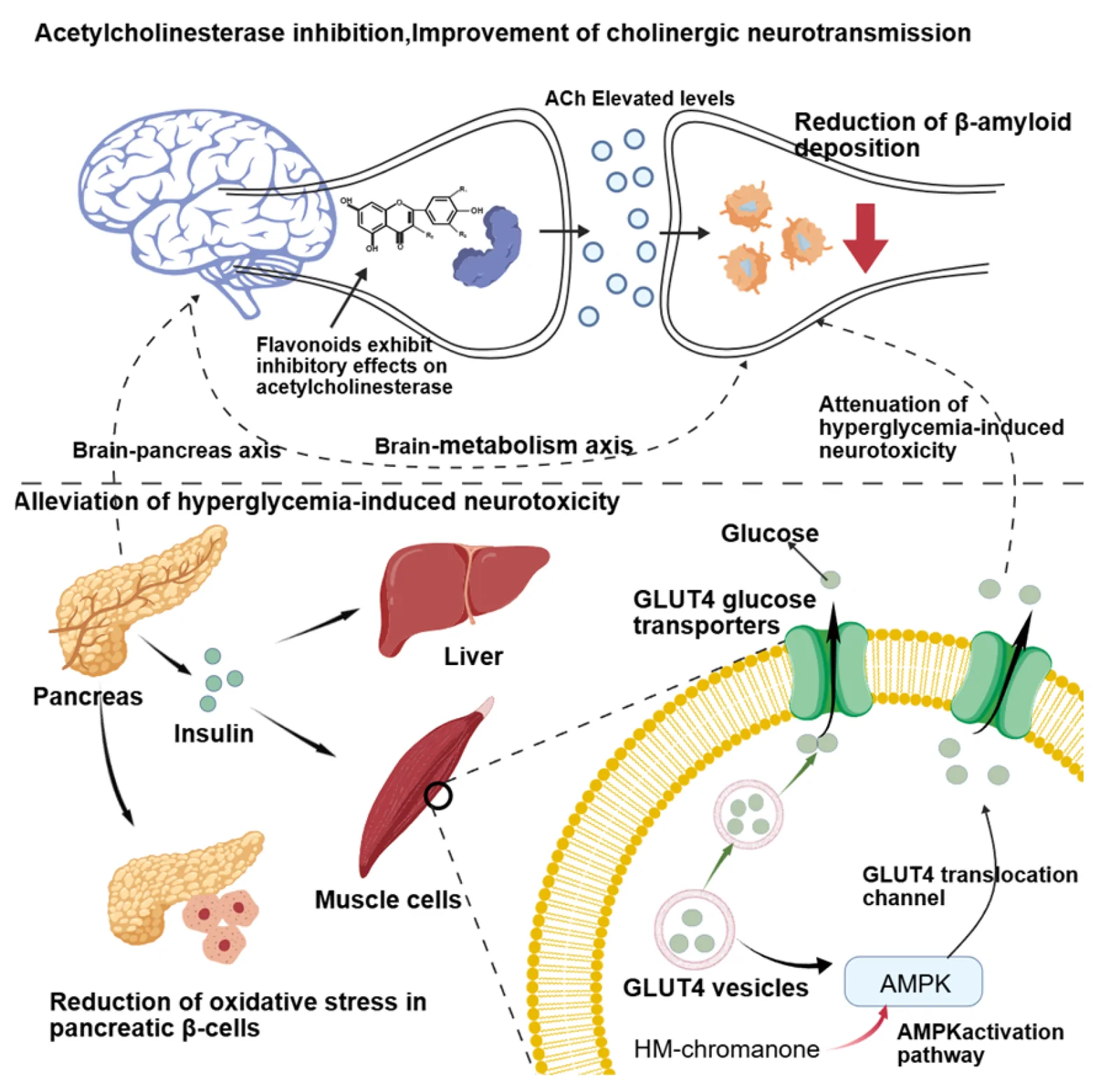
The neuroprotection and hypoglycemia effects of *Portulacae Herba.* Flavonoids from *Portulaca oleracea* L. inhibit acetylcholinesterase activity, elevate the concentration of brain acetylcholine (ACh), and reduce β-amyloid deposition (red downward arrow). Through the brain-pancreas axis and brain-metabolism axis, *Portulaca oleracea* alleviates hyperglycemia-induced neurotoxicity. It reduces oxidative stress in pancreatic β-cells and promotes insulin secretion. HM-chromanone activates the AMPK pathway to trigger GLUT4 translocation in muscle cells, which enhances glucose uptake and clearance, and ameliorates high-glucose-mediated neuronal injury.Abbreviations: ACh, acetylcholine; AMPK, adenosine 5’-monophosphate-activated protein kinase; GLUT4, glucose transporter 4.

Purslane polysaccharides, alkaloids, and flavonoids are the key hypoglycemic components that can regulate blood glucose levels through multiple mechanisms and improve diabetic complications [120]. Purslane polysaccharides can increase insulin levels, lower fasting blood glucose, and improve glucose metabolism disorders in diabetic mice; their decoction preparation can effectively alleviate pathological damage in diabetic rats [121]. Quercetin and kaempferol glycosides competitively inhibit α-glucosidase, thereby blocking the breakdown and absorption of carbohydrates; active components in purslane can also modulate K_ATP channels to regulate insulin secretion in pancreatic β-cells [122]. The alkaloids in purslane promote insulin secretion and accelerate glycogen synthesis, while polysaccharides help regulate blood glucose levels through their antioxidant effects. Its seeds enhance insulin sensitivity, exhibiting a hypoglycemic effect superior to metformin, while also improving diabetic vascular damage and symptoms of gastroparesis [123]. Furthermore, purslane can alleviate inflammatory damage associated with diabetes by inhibiting the NF-κB pathway and achieve dual regulation of blood glucose and lipid levels via the AMPK/PPAR-γ/GLUT4 pathway [124,125]. It can also improve glucose metabolism disorders by regulating the gut microbiota and repairing the intestinal barrier [126] (Figure 4).

##### Antitumor Effects

Components of purslane, such as polysaccharides, flavonoids, alkaloids, and seed oil, exhibit significant antitumor activity and can inhibit tumor proliferation through mechanisms including multi-target regulation, induction of apoptosis and autophagy, and targeted drug delivery [127]. Studies have confirmed that purslane polysaccharides can inhibit liver cancer cell proliferation and induce tumor apoptosis in vitro, and can suppress the growth of transplanted tumors in mice in vivo [128]. They exert their antitumor effects by blocking tumor proliferation, inhibiting angiogenesis, and reshaping immune surveillance [129]; the structural characteristics of the polysaccharides directly influence their efficacy, and they can also specifically regulate the TLR4 pathway to effectively inhibit the growth of cervical cancer xenografts [130]. Purslane alkaloids can block the nutrient supply to tumors; brachygenin A can inhibit the migration of cervical cancer cells; and purslane seed oil can broadly inhibit the proliferation of various tumor cells [131].

##### Digestive System Regulation

Purslane protects the digestive system by repairing the intestinal barrier, restoring the gut microbiota, suppressing intestinal inflammation, and blocking the inflammatory-to-cancer transition [132,133]. In vivo studies have demonstrated that purslane granule preparations markedly reduce the incidence of bacterial diarrhea in mice and accelerate the recovery of intestinal function [134,135]. Its aqueous extract can optimize the structure of the gut microbiota, increase the abundance of beneficial bacteria, regulate the metabolic homeostasis of amino acids and bile acids, and effectively alleviate symptoms of diarrhea [136]. Purslane polysaccharides ameliorate gut microecological dysbiosis, enhance intestinal mucosal immune responses, reduce endotoxin accumulation, and exert therapeutic effects against ulcerative colitis [75]. Animal experiments have confirmed that purslane polysaccharides attenuate colonic pathological damage in a dose-dependent manner and comprehensively restore intestinal integrity via multiple mechanisms, including repairing the intestinal barrier, expanding beneficial bacterial populations, and promoting short-chain fatty acid production [80,137]. Furthermore, these polysaccharides modulate inflammatory signaling pathways to mitigate intestinal inflammatory injury and block the inflammation-to-carcinogenesis signaling axis, thereby suppressing the progression from chronic colitis to colorectal cancer [138]. To date, research in this domain has been largely confined to ulcerative colitis and bacterial diarrhea, whereas investigations into other digestive disorders such as gastric ulcers and irritable bowel syndrome remain relatively limited [139].

### 3.3. Applications in Veterinary Clinical Practice

Throughout this section, evidence is classified by study type (controlled trial, field observation, or in vitro study), and where purslane was administered as part of a combination, fermented preparation, or multi-component intervention, this is explicitly stated.

#### 3.3.1. Poultry

Purslane is often used in the poultry industry as a new type of green starter medication; it can improve the spleen and bursa of Fabricius indices in chickens and enhance the body’s immune function and disease resistance [140]. When processed using fermentation technology, it is effective in preventing and treating white diarrhea in chickens [141]; however, this evidence pertains to the fermented preparation rather than to unprocessed *P. oleracea* itself. In a controlled trial, dietary supplementation with dried purslane powder at 2% of the diet for 42 days significantly increased plasma and hepatic peroxidase activity, enhanced antioxidant capacity, reduced the feed-to-weight ratio, and increased daily weight gain [142]. In another controlled trial, dried purslane powder at 2–3% of the diet over a 42-day feeding period effectively improved overall growth performance in broilers [143]. In a field observation, feeding fresh, chopped purslane (ad libitum) as a supplement promoted feed intake and feeding rate, maintained normal fecal consistency, and reduced production costs [144]. In a controlled trial in layer chickens, dietary supplementation with 20 g/kg of dried purslane powder for 8 weeks significantly increased the omega-3 polyunsaturated fatty acid content in eggs, while also improving laying rate and enhancing disease resistance [145].

#### 3.3.2. Livestock

In swine production, dietary supplementation with 2% purslane powder for 28 days has been reported to improve growth performance in weaned piglets [146,147]. Additionally, purslane polysaccharides have demonstrated in vitro antiviral activity against porcine epidemic diarrhea virus (PEDV) at concentrations ranging from 6.25 to 25.00 μg/mL [148], although in vivo efficacy has not been established. Traditional use for weaning diarrhea, gastroenteritis, and dysentery in piglets has been documented [100,149], but controlled clinical trials validating these effects in pigs are scarce.

In cattle, one in vitro study reported that a water extract of purslane inhibited major mastitis-associated pathogens, including *Escherichia coli*, *Staphylococcus aureus*, and *Streptococcus agalactiae* [150]. However, no controlled clinical trials evaluating the efficacy of purslane for treatment of bovine mastitis or other conditions (e.g., stomatitis, diarrhea) have been identified. Traditional use for stomatitis and bloody diarrhea has been noted [100], but these reports lack rigorous experimental validation.

#### 3.3.3. Companion Animals (Current Evidence and Potential Applications)

To date, the direct evidence supporting the use of purslane in companion animal species is extremely limited. Only one controlled study has been reported in dogs: combined administration of fermented purslane and probiotics isolated from feces of healthy dogs effectively alleviated clinical signs of senna-induced diarrhea in canines [151]. Because the intervention was a combined fermented purslane–probiotic preparation, the observed effect cannot be attributed specifically to *P. oleracea*. Beyond this single study, no direct experimental or clinical evidence exists in dogs or cats regarding the effects of purslane on digestive, cardiovascular, metabolic, or age-associated disorders. Any claims regarding these potential applications in companion animals would be purely extrapolated from in vitro studies, rodent models, or human clinical trials, and such extrapolation is not scientifically justified without species-specific validation. Importantly, dogs and cats should not be treated as a single biological category for the purpose of pharmacological or nutritional recommendations; their metabolic responses to phytochemicals differ substantially. Future research should prioritize controlled trials in companion animal species before any clinical recommendations can be made.

Table 5 summarizes veterinary applications of *purslane* (*Portulaca oleracea* L.), including its preparations, dosages, administration routes, and primary outcomes in target animal species.

## 4. Discussion

This narrative review systematically synthesizes the available evidence on the chemical constituents, pharmacological activities, and veterinary clinical applications of purslane. Through comprehensive collation and integration of the 160 eligible studies, several key observations and overarching patterns emerge, alongside important knowledge gaps that warrant future investigation.

Integrated Findings and Pharmacological Differentiation. One of the most salient findings from this synthesis is the distinct pharmacological preference exhibited by different classes of bioactive compounds within purslane. Specifically, polysaccharides predominantly exert immunomodulatory, hepatoprotective, hypoglycemic, and antitumor activities, largely through the modulation of immune cell function (via the TLR4/MyD88 pathway) and activation of the AMPK signaling axis [32,126,129]. In contrast, flavonoids, particularly quercetin and kaempferol glycosides, are primarily responsible for the anti-inflammatory, antioxidant, and neuroprotective effects, acting through the scavenging of free radicals and inhibition of the NF-κB/MAPK pathways [6,9,73]. Alkaloids contribute notably to insulin secretion enhancement and inflammatory relief [21,30], whereas unsaturated fatty acids (especially omega-3 series) function chiefly in lipid regulation and cardiovascular protection [37,75]. These compound-specific activity profiles may provide a rationale for future studies evaluating polysaccharide-enriched preparations in weaned piglets or flavonoid-rich fractions in poultry inflammatory models. Such differentiation could be explored in veterinary research, where species-specific metabolic responses to phytochemicals may vary considerably [100,152].

Evidence Gaps and Methodological Limitations. Despite the wealth of in vitro and animal model data, significant translational gaps persist. First, the majority of pharmacological studies are conducted in rodent models (mice and rats) or in vitro cell lines, with limited direct validation in target veterinary species (swine, poultry, or cattle). While a growing number of poultry and swine trials have been reported [149,150], pharmacokinetic profiles—including absorption, distribution, metabolism, and excretion—of purslane bioactive compounds in these production animals remain poorly characterized. Second, dose–response relationships in target species are inadequately defined. Although some studies report quantitative data (e.g., MICs of 0.5–1.0 mg/mL for MRSA, or dietary supplementation of 2–3% in broilers), standardized dosing protocols across studies are lacking, making direct comparisons and clinical guideline development challenging. Third, the antitussive and bronchodilatory effects, although widely cited, are largely inferred from composite herbal formulations rather than from isolated purslane extracts, and mechanistic studies targeting β_2_-adrenergic or muscarinic receptors in large animal models are conspicuously absent [66,68]. Fourth, long-term safety data and potential herb–drug interactions have not been systematically evaluated in veterinary contexts. Residue studies in edible tissues and milk are also scarce, which is a critical consideration for food-producing animals [154]. Fifth, most digestive system studies are confined to ulcerative colitis and bacterial diarrhea, with limited investigation into other common disorders such as gastric ulcers, pancreatitis, or irritable bowel syndrome in companion animals [143]. Addressing these gaps will require coordinated efforts between pharmacologists, veterinary clinicians, and analytical chemists to develop species-relevant experimental models and analytical methods.

Safety Concerns and Toxicological Considerations. Despite the promising pharmacological activities discussed above, the safety profile of purslane for veterinary applications remains critically under-investigated. Two primary toxicological concerns warrant particular attention: oxalate content and nitrate accumulation. Purslane is known to contain high levels of oxalates, with reported total oxalate content reaching up to 10.75% on a dry matter basis in some field specimens [157], and generally ranging from 1.3% to as high as 17% dry matter [158]. Field and experimental evidence has documented acute oxalate poisoning in sheep and cattle following ingestion of purslane. Clinical cases include a flock of 2100 ewes of which five died within hours after being released into a paddock rich in purslane, with autopsy revealing high oxalate levels (10.75%) in the ingested plant material and 4.77% in rumen contents [157]. Oxalates can chelate calcium, leading to hypocalcemia and the deposition of calcium oxalate crystals in renal tubules, potentially causing acute renal failure in susceptible animals. Ruminants and equines are particularly sensitive to oxalate poisoning [158]. Nitrate accumulation represents a second significant safety concern. Purslane has a well-documented capacity to accumulate nitrate in its leaf tissue, particularly under high nitrogen fertilization regimes [158]. Reported nitrate concentrations in purslane leaves can reach 48.98 mg/g dry weight [158]. A recent veterinary report documented an acute outbreak of nitrate/nitrite poisoning in feedlot calves, affecting 17.5% and 5.7% of 8-month-old calves in two pens, respectively, due to consumption of purslane present in the pens. Post-mortem examination revealed abundant purslane stems in the rumen content, and high nitrate levels were detected in purslane samples collected from the affected pens [159]. Nitrate is reduced to nitrite by ruminal microorganisms, and absorbed nitrite oxidizes hemoglobin to methemoglobin, resulting in tissue hypoxia and potentially death. A case of nitrite poisoning in yellow cattle following purslane consumption has also been reported in China. The absence of reported toxicity in short-term rodent studies does not equate to safety in target veterinary species. The variability of oxalate and nitrate content in purslane depends on multiple factors, including genotype, growth stage, soil composition, nitrogen fertilization, and environmental conditions [159]. Processing methods such as boiling or steaming can reduce oxalate content, but such treatments are generally not applicable to large-scale feed applications. Furthermore, long-term feeding studies, reproductive toxicity assessments, potential herb–drug interactions, and residue depletion studies in edible tissues and milk are completely absent from the current literature. Until such data become available, the use of purslane in food-producing animals should be approached with caution, and nitrate testing of forage material is strongly recommended before large-scale feeding.

Implications for Veterinary Practice and Future Directions. From a practical standpoint, the existing evidence supports further investigation of purslane as a promising candidate for antibiotic-sparing strategies that may contribute to reducing reliance on conventional growth-promoting antibiotics in poultry and swine production, particularly given its broad-spectrum antibacterial and immunomodulatory properties [80,82,84]. However, this evidence should not be interpreted as demonstrating an actual reduction in antimicrobial requirement or use. Any consideration of large-scale use in food-producing animals must be preceded by species-specific safety and residue-depletion data. Reported improvements in feed conversion ratio and reductions in post-weaning diarrhea in some purslane-feeding studies align well with global efforts to reduce antimicrobial use in livestock production [147,150]. Furthermore, the enrichment of omega-3 fatty acids in eggs and meat from purslane-fed poultry may offer an added nutritional benefit that could enhance the market value of animal products [149]. However, successful translation requires the development of standardized, quality-controlled extracts with well-defined active marker compounds. Several recent studies have emphasized the importance of extraction methods and quality control parameters in determining the consistency of pharmacological outcomes [31,50], yet no universally accepted quality standard exists for veterinary-grade purslane preparations. Future research should prioritize: (i) species-specific pharmacokinetic and pharmacodynamic studies in pigs, poultry, and dogs to establish optimal dosage regimens; (ii) large-scale, randomized controlled field trials with clearly defined clinical endpoints (e.g., mortality, weight gain, fecal consistency scores) to substantiate efficacy claims; (iii) elucidation of receptor-level mechanisms using target-specific antagonists and gene-knockout models, particularly for the anti-inflammatory, hypoglycemic, and neuroprotective pathways; (iv) investigation of potential synergistic or antagonistic interactions among different phytochemicals when used in combination; and (v) systematic safety evaluations, including toxicological studies and residue analysis in animal-derived food products, to ensure consumer safety and regulatory compliance. Until species-specific safety and residue-depletion data become available, large-scale use of purslane in food-producing animals should be approached with caution.

## 5. Conclusions

In conclusion, purslane is a phytochemically diverse plant with promising pharmacological activities and potential for further development as a functional feed ingredient or source of compounds for veterinary research. Its diverse bioactive constituents confer a wide spectrum of beneficial effects, ranging from antimicrobial and anti-inflammatory to metabolism-regulating and immunomodulatory activities. Nevertheless, the current evidence base is predominantly preclinical, and rigorous, species-specific clinical trials are urgently needed to translate these promising findings into evidence-based veterinary practice. Until such data become available, the implementation of purslane-based interventions in routine animal healthcare should be approached with careful attention to extract standardization, dosage optimization, and quality control to ensure both safety and efficacy.

## Figures and Tables

**Table 1:** Flavonoids in *Portulacae Herba*.

No.	Compound Name	Molecular Formula	Ref.
1	Quercetin	C_15_H_10_O_7_	[5]
2	Kaempferol	C_15_H_10_O_6_	[8,12]
3	Apigenin	C_15_H_10_O_5_	[6]
4	Luteolin	C_15_H_10_O_6_	[13]
5	Portulal A	C_20_H_32_O_4_	[14]
6	3-Hyroxy-5, 6-epoxy-β-ionone	C_13_H_20_O_3_	[15]
7	Genistein	C_15_H_10_O_5_	[15]
8	Hesperidin	C_28_H_34_O_15_	[6,16]

**Table 2:** Alkaloids in *Portulacae Herba*.

No.	Compound Name	Molecular Formula	Ref.
1	Norepinephrine	C_8_H_11_NO_3_	[19]
2	Dopa	C_9_H_11_NO_4_	[20]
3	Dopamine	C_8_H_11_NO_2_	[20]
4	Adenosine	C_10_H_13_N_5_O_4_	[21]
5	Allantoin	C_4_H_6_N_4_O_3_	[22]
6	Portulacamide A	C_27_H_53_NO_5_	[21]
7	Oleraciamide A	C_17_H_26_N_2_O_3_	[23]
8	Oleraciamide B	C_17_H_26_N_2_O_4_	[23]
9	Oleracimine	C_18_H_26_N_2_O	[24]

**Table 3:** Organic acids in *Portulacae Herba*.

No.	Compound Name	Molecular Formula	Ref.
1	Malic acid	C_4_H_6_O_5_	[31]
2	Oxalic acid	C_2_H_2_O_4_	[32]
3	Citric acid	C_6_H_8_O_7_	[33]
4	Vanillic acid	C_8_H_8_O_4_	[31]
5	Docosahexaenoic acid	C_22_H_32_O_2_	[31]
6	Erucic acid	C_22_H_42_O_2_	[9]
7	Arachidonic acid	C_20_H_32_O_2_	[34]
8	Eicosapentaenoic acid	C_20_H_30_O_2_	[34]
9	Succinic acid (butanedioic acid)	C_4_H_6_O_4_	[30]
10	Caffeic acid	C_9_H_8_O_4_	[35]
11	Ferulic acid	C_10_H_10_O_4_	[36]
12	Pyroglutamic acid	C_5_H_7_NO_3_	[31]

**Table 4:** Alcohols, esters, sterols, and terpenoids in *Portulacae Herba*.

No.	Compound Name	Molecular Formula	Class	Ref.
1	Lilacin	C_17_H_24_O_9_	Alcohol glycoside	[60]
2	Lupinol	C_30_H_50_O	Triterpenoid	[57]
3	Linoleic acid triglyceride	C_57_H_98_O_6_	Triglyceride (ester)	[19]
4	β-Sitosterol	C_29_H_50_O	Phytosterol	[57]
5	β-Carotene	C_40_H_56_	Tetraterpenoid (Carotenoid)	[32]
6	Inositol	C_6_H_12_O_6_	Cyclitol (alcohol)	[59]
7	α-Farnesol	C_15_H_26_O	Sesquiterpene	[59]
8	β-Farnesol	C_15_H_26_O	Sesquiterpene	[59]
9	Cycloartanol	C_30_H_52_O	Triterpenoid	[59]
10	n-Tridecanol	C_13_H_28_O	Aliphatic alcohol	[59]
11	α-Palmitic acid monoglyceride	C_19_H_38_O_4_	Ester	[21]
12	Portulacerebroside A	C_48_H_93_NO_10_	Cerebroside	[60]
13	Brassinosterol	C_28_H_48_O_6_	Sterol	[61]

**Table 5:** Summary of veterinary applications of *purslane* (*Portulaca oleracea* L.): preparation, dosage, administration, and main outcomes in target animal species.

Species	Preparation	Dose/Inclusion	Route	Duration	Key Findings	Study Type	Ref.
Broiler	Dried powder	2% of diet	Dietary	42 d	↑ plasma and hepatic peroxidase activity, ↑ antioxidant capacity, ↓ feed-to-weight ratio, ↑ daily weight gain	Controlled trial	[142]
Broiler	Dried powder	2–3% of diet	Dietary	42 d	↑ overall growth performance	Controlled trial	[143]
Broiler	Fresh, chopped	Ad libitum supplement	Dietary	Not specified	↑ feed intake and feeding rate, maintained normal fecal consistency, ↓ production costs	Field observation	[144]
Broiler	Dried powder	1% of diet	Dietary	42 d	↑ body weight gain, ↑ European Production Efficiency Factor, ↑ antibody titer against IBDV (* *p* * < 0.05)	Randomized controlled trial	[152]
Broiler	Methanol extract	150 or 300 mg/kg	Dietary	42 d	↓ negative impacts of T_3_-induced ascites, including right ventricular hypertrophy and mortality	Controlled trial	[153]
Layer	Dried powder	20 g/kg (2%)	Dietary	8 wk	↑ egg ω-3 PUFA content (C18:3ω-3 and C22:6ω-3, * *p* * < 0.05), ↑ laying rate, ↑ disease resistance	Controlled trial	[145]
Wenchang chicken	Extract (POE)	0.2% or 0.4% of diet	Dietary	Not specified	↑ growth performance, ↑ immune function, ↑ meat quality	Controlled trial	[154]
Wenchang chicken	Extract	250 mg/kg	Dietary	Not specified	↑ intestinal function, modulated gut microbiota against Salmonella pullorum	Controlled trial	[155]
Weaned piglet	Dried powder	2% of diet	Dietary	28 d	↑ growth performance	Controlled trial	[146]
Weaned piglet	Fermented purslane	0.20% of diet	Dietary	28 d	↑ ADG (first 14 d), ↓ diarrhea occurrence (d 10–13, * *p* * < 0.05), ↓ serum IL-6 (d 14 and 28), ↑ serum SOD, improved gut integrity	Randomized controlled trial	[156]
Piglet	Polysaccharide	6.25–25.00 μg/mL	In vitro	—	Antiviral activity against porcine epidemic diarrhea virus (PEDV) in vitro; in vivo efficacy not established	In vitro study	[100]
Dairy cow	Aqueous extract	0.12–0.5 g/mL	In vitro	—	Inhibited mastitis pathogens (*E. coli*, *S. aureus*, *S. agalactiae*); zone of inhibition 13.8–18 mm	In vitro study	[148]
Dairy cow	Ethanolic extract	0.12–0.5 g/mL	In vitro	—	Higher antibacterial activity than aqueous extract (except against *E. coli*); zone of inhibition 14.8–19.6 mm	In vitro study	[148]
Dog	Fermented purslane + probiotics	Combined preparation	Oral	Not specified	Alleviated clinical signs of senna-induced diarrhea; superior to probiotic or purslane alone	Controlled trial	[151]

Abbreviations: ADG, average daily gain; IBDV, infectious bursal disease virus; IL-6, interleukin-6; POE, *Portulaca oleracea* extract;PUFA, polyunsaturated fatty acid; SOD, superoxide dismutase; T_3_, triiodothyronine.

## Data Availability

No new data were created or analyzed in this study. Data sharing is not applicable to this article.

## Supplementary Materials

The following supporting information can be downloaded at: https://www.mdpi.com/article/10.3390/vetsci13101017/s1, Table S1: Full database-specific search strategies.
