# Supplementary material for: Research Progress on the Chemical Composition, Pharmacological Effects and Veterinary Clinical Application of Purslane (Portulaca oleracea L.)
Source: Vet Sci. 2026 Sep 26;13(10):1017. doi: 10.3390/vetsci13101017 (PMC13709476; doi:10.3390/vetsci13101017)
Supplement: Supplementary file 1 [file vetsci-13-01017-s001.zip › vetsci-4544501-supplementary.pdf]

Supplementary Table S1

Table S1. Full database-specific search strategies.

Theme	Database	Search strategy	Records retrieved
Reviews and ethnopharmacology	PubMed	("Portulaca oleracea" [tiab] OR purslane [tiab]) AND (review [pt] OR review [tiab] OR ethnopharmacology [tiab] OR "research progress" [tiab] OR overview [tiab])	64
	CNKI	(SU%=Portulaca oleracea OR SU%=Portulaca) AND (TI=review OR KY=review OR TI=research progress OR KY=research progress OR TI=ethnopharmacology OR KY=ethnopharmacology)	93
Chemical composition, extraction, and analysis	PubMed	("Portulaca oleracea" [tiab] OR purslane [tiab]) AND ("chemical constituents" [tiab] OR isolation [tiab] OR identification [tiab] OR extraction [tiab] OR polysaccharide [tiab] OR alkaloid [tiab] OR flavonoid [tiab] OR "organic acid" [tiab] OR HPLC [tiab] OR "GC-MS" [tiab])	214
	CNKI	(SU%=Portulaca oleracea OR SU%=Portulaca) AND (TI=chemical constituents OR KY=chemical constituents OR TI=isolation and identification OR KY=isolation and identification OR TI=extraction process OR KY=extraction process OR TI=polysaccharide OR KY=polysaccharide OR TI=alkaloid OR KY=alkaloid OR TI=flavonoid OR KY=flavonoid OR TI=organic acid OR KY=organic acid OR TI=HPLC OR KY=HPLC OR TI=GC-MS OR KY=GC-MS)	525
Pharmacological activities and mechanisms	PubMed	("Portulaca oleracea" [tiab] OR purslane [tiab]) AND ("anti-inflammatory" [tiab] OR immunomodulatory [tiab] OR antioxidant [tiab] OR antibacterial [tiab] OR antimicrobial [tiab] OR antitumor [tiab] OR anticancer [tiab] OR neuroprotective [tiab] OR hypoglycemic [tiab] OR antidiabetic [tiab] OR hypolipidemic [tiab] OR hepatoprotective [tiab] OR "intestinal" [tiab] OR mechanism [tiab])	463
	CNKI	(SU%=Portulaca oleracea OR SU%=Portulaca) AND (TI=anti-inflammatory OR KY=anti-inflammatory OR TI=immunomodulatory OR KY=immunomodulatory OR TI=antioxidant OR KY=antioxidant OR TI=antibacterial OR KY=antibacterial OR TI=antitumor OR KY=antitumor OR TI=neuroprotective OR KY=neuroprotective OR TI=hypoglycemic OR KY=hypoglycemic OR TI=hypolipidemic OR KY=hypolipidemic OR TI=hepatoprotective OR KY=hepatoprotective OR TI=intestinal OR KY=intestinal OR TI=mechanism OR KY=mechanism)	362
Agriculture, food processing, feed, and toxicology	PubMed	("Portulaca oleracea" [tiab] OR purslane [tiab]) AND (cultivation [tiab] OR agronomic [tiab] OR harvest [tiab] OR processing [tiab] OR "seed oil" [tiab] OR feed [tiab] OR broiler [tiab] OR piglet [tiab] OR toxicity [tiab] OR oxalate [tiab] OR nitrate [tiab])	137
	CNKI	(SU%=Portulaca oleracea OR SU%=Portulaca) AND (TI=cultivation OR KY=cultivation OR TI=agronomy OR KY=agronomy OR TI=harvest OR KY=harvest OR TI=processing OR KY=processing OR TI=seed oil OR KY=seed oil OR TI=feed OR KY=feed OR TI=broiler OR KY=broiler OR TI=piglet OR KY=piglet OR TI=toxicology OR KY=toxicology OR TI=oxalate OR KY=oxalate OR TI=nitrate OR KY=nitrate)	247

Note: Search date for all databases: May 2026. No language filter was applied at the search stage; non-English and non-Chinese records were excluded during screening. CNKI search strings were originally executed in Chinese and are presented here in English translation for consistency.
